# Metallothionein 2A core promoter region genetic polymorphism and its impact on the risk, tumor behavior, and recurrences of sinonasal inverted papilloma (Schneiderian papilloma)

**DOI:** 10.1007/s13277-015-3616-7

**Published:** 2015-06-03

**Authors:** Katarzyna Starska, Magdalena Bryś, Ewa Forma, Jurek Olszewski, Piotr Pietkiewicz, Iwona Lewy-Trenda, Olga Stasikowska-Kanicka, Marian Danilewicz, Anna Krześlak

**Affiliations:** 1 I Department of Otolaryngology and Laryngological Oncology, Medical University of Łódź, Kopcinskiego 22, 90-153 Łódź, Poland; 2 Department of Cytobiochemistry, University of Łódź, Pomorska 142/143, 90-236 Łódź, Poland; 3 II Department of Otolaryngology and Laryngological Oncology, Medical University of Łódź, Żeromskiego 113, 90-549 Łódź, Poland; 4 Department of Pathology, Medical University of Łódź, Pomorska 251, 92-213 Łódź, Poland

**Keywords:** Metallothionein 2A (MT2A), Single-nucleotide polymorphism (SNP), Krouse staging system, Recurrences, IP, Sinonasal inverted papilloma (Schneiderian papilloma)

## Abstract

Inverted papillomas are a unique group of locally aggressive benign epithelial neoplasms in the nasal cavity and paranasal sinuses arising from the Schneiderian mucosa. Metallothioneins are sulfhydryl-rich heavy metal-binding proteins required for metal toxicity protection and regulation of biological mechanisms including proliferation and invasion. The goal of this study was to identify three SNPs at loci −5 A/G (rs28366003) and −209 A/G (rs1610216) in the core promoter region and at locus +838 C/G (rs10636) in 3′UTR region of the *MT2A* gene with IP risk and with tumor invasiveness according to Krouse staging. Genotyping was performed using the PCR restriction fragment length polymorphism technique in 130 genetically unrelated IP individuals, and 418 randomly selected healthy volunteers. The presence of the rs28366003 SNP was significantly related to the risk of IP within the present population-based case-control study. Compared to homozygous common allele carriers, heterozygosity and homozygosity for the G variant had a significantly increased risk of IP (adjusted odds ratio [OR] = 7.71, 95 % confidence interval [CI]: 4.01–14.91, *p*_dominant_ < 0.001). Moreover, risk allele carriers demonstrated higher Krouse stage (pT1 vs. pT2-4) (OR = 19.32; 95 % CI, 2.30–173.53; *p* < 0.0001), diffuse tumor growth (OR = 4.58; 95 % CI, 1.70–12.11; *p* = 0.0008), bone destruction (OR = 4.13; 95 % CI, 1.50–11.60; *p* = 0.003), and higher incidence of tumor recurrences (OR = 5.11; 95 % CI, 1.68–15.20; *p* = 0.001). The findings suggest that *MT2A* gene variation rs28366003 may be implicated in the etiology of sinonasal inverted papilloma in a Polish population.

## Introduction

Sinonasal inverted papillomas (Schneiderian papillomas) of the nasal cavity and paranasal sinuses are unique, locally aggressive, primarily benign epithelial neoplasms derived from the Schneiderian membrane with an endophytic growth pattern and proliferation in the underlying stroma. The characteristic attributes of these head and neck tumors are local invasiveness and aggressive growth with local bone destruction and a higher tendency to recur, resulting in appropriate clinical problems. Moreover, once epithelial dysplasia in IP tissues occurs, metaplasia and malignant transformation into carcinoma in situ or invasive squamous cell carcinoma may follow [1, 2]. Unfortunately, although a range of possible etiologies including possible viral (HPV infection), inflammatory, environmental, and occupational etiologies have been proposed, no conclusive basis has yet been identified for the development and pathogenesis of Schneiderian papillomas [3, 4].

The metallothioneins (MTs) family of low molecular mass (6–7 kDa) proteins is a family of metal-binding and metal-absorbing, cysteine-rich molecules which affect the homeostasis of intracellular metals such as cadmium, lead, mercury, zinc, and copper [5–7]. MTs can react with heavy and trace metal ions, thus regulating intra- and extracellular distribution of the metal and its donation to various crucial transcription factors and enzymes. Hence, MTs are involved in many pathophysiological processes including metal detoxification, cell proliferation, apoptosis, and deactivation of reactive oxygen species [5–8]. The transcription of the MT gene is activated in response to a wide range of stimuli, including oxidative stress-inducing agents, cytokines, hormones and various chemical agents, as well as Cd and Zn in particular [5, 6, 9, 10]. A crucial role in the activation of the metal-induced MT promoter region is played by the six zinc-finger-metal-responsive transcription factor (MTF-1) and short *cis*-acting DNA metal response elements (MREs) as transcriptional activators and a nuclear factor I (NF-I) protein as a downregulator of MT expression [11]. MTF-1 also exerts effects on MT gene transcription by independent increase in MTF-1 DNA-binding activity [12, 13].

Eleven functional human MT isoforms have been identified so far. Among them, MT1/MT2 isoforms are the most active in human cells and may participate as tumor regulators in such crucial mechanisms as inhibition of NF-κB signaling, modulation of p53 zinc-dependent activity, as well as regulation of the PIK3/AKT and Rb/E2F pathways [9, 10, 14–17]. In the last years, several association studies have been performed which indicate that metallothioneins may be implicated in the development of various types of human neoplastic diseases and determine the clinical course of many tumors, e.g., larynx, breast, kidney, lung, ovary, prostate, testis, urinary bladder, cervical or pancreatic cancers [5, 9, 10, 14, 17–19]. Due to the multidirectional nature of intracellular MT activity and possible participation in such crucial tumorigenic processes as differentiation, proliferation, angiogenesis and response to oxidative stress species, many researchers examine its relationship as a potential biomarker with the etiology, progression and prognosis of various tumors, but with ambiguous and often conflicting results [5–7, 9, 10, 14, 17, 18, 20].

Recently, an individual genetic variation identified in the core promoter region of MTs and the diverse frequency of essential MT alleles, in particular MT2A, was shown to be associated with the risk of various human tumors in some ethnic populations [5, 10, 18, 20–23]. The contribution of single-nucleotide polymorphisms (SNPs) in the *MT2A* gene to variation in the cellular activities of metallothioneins and the signaling of MT-dependent pathways may steer the enhancement of tumor development and growth toward increased DNA damage, enhanced genomic instability, deregulated cell proliferation, inhibited apoptosis, and induced oxidative stress [6, 8]. Thus, in the last half decade, research has been focused on the identification of the aftermath and the mechanisms through which the presence of SNPs in some genes, inter alia the *MT2A* gene, may induce a malignant cell phenotype and determine cell proliferation, growth and tumor invasion. However, such individual studies concern only neoplasms of the head and neck region in such locations as the nasopharynx, salivary glands, tongue, or larynx [10, 24–27]. Although the data indicates that the presence of SNPs in MT genes has an impact on MT1/MT2 expression, and that the occurrence of different genotypes are associated with cancer risk, tumor behavior, and chemotherapy resistance in head and neck cancers, these findings have not been unambiguously confirmed, and other studies suggest a range of final conclusions [10, 24–27]. Despite conflicting data, most research indicates that changes identified in the core promoter region in the *MT2A* gene near the TATA box may be considered as novel biomarkers of risk and tumor invasiveness, as well as a potential new therapeutic targets for treatment strategies to modify or inhibit neoplastic development and progression of various types of tumors [5, 10, 18, 20–23].

Unfortunately, a literature survey reveals no publication describing the activity of *MT2A* isoforms and their relationship with the risk and tumor clinical behavior in sinonasal Schneiderian papillomas. Moreover, the etiology of this disease is largely unknown, although epidemiological studies suggest the involvement of viral, immunologic and occupational factors. Therefore, more research is needed to better understand the possible biological mechanisms of development and the potential role of both MT2A expression and genetic polymorphism in the *MT2A* gene core promoter region in this rare, locally aggressive tumorigenic process.

The present population-based case-control study was aimed at investigating the genetic association between the risk of sinonasal inverted papilloma (Schneiderian papilloma) in inhabitants of Poland, and the presence of three selected single-nucleotide polymorphisms: at loci −5 A/G (rs28366003) and −209 A/G (rs1610216) in the core promoter region and at locus +838 C/G (rs10636) in 3′UTR region of the *MT2A* gene. In addition, the effect of these SNPs on allele-specific gene expression and invasiveness of neoplastic process according to Krouse staging and incidence of recurrences has been identified.

## Materials and methods

### Study population and lifestyle risk factors

In this study, 130 tissue samples, including sinonasal inverted papilloma (Schneiderian papilloma) cases from genetically unrelated individuals (89 male and 41 female, mean age 59.14 ± 12.66 years), recruited between January 2003 and December 2014, were selected and investigated. The patients were under treatment at the I and II Departments of Otolaryngology and Laryngological Oncology, Medical University of Łódź, Poland. All patients had received a confirmed diagnosis of inverted papilloma (IP) based on histopathological evaluation and had undergone functional endoscopic sinonasal surgery (FESS), although this was dependent on the extent of neoplastic lesions described by CT scans of nasal cavity and paranasal sinuses performed before surgery and assessed during an earlier FESS procedure. The observation period after surgical treatment ranged from 5 to 154 months (mean period 59.37 ± 37.63 months). Recurrence occurred after FESS in 18 (13.8 %) cases in the group studied. Sinonasal squamous cell carcinoma (SCC) associated with IP (synchronous carcinoma) was found in one case in group studied. The IP samples were collected from the tumor localized in nasal cavities and/or paranasal sinuses. The criteria for patient participation in this study were as follows: (1) a pathologically confirmed diagnosis of sinonasal inverted papilloma (Schneiderian papilloma), (2) a negative history of previously diagnosis with other types of primary cancers, (3) a negative history of receiving prior immuno-, radio- or chemotherapy due to other types of primary neoplasms. The control group comprised 418 randomly selected healthy volunteers in the same age group (314 male and 104 female, mean age 64.17 ± 8.28 years) as previously described [19]: all controls were non-related individuals who had never been diagnosed with head and neck tumors nor any other neoplasms, nor had they received prior immuno-, radio- or chemotherapy for other reasons. All of the studied individuals, patients and controls were Caucasians from the same ethnic and geographical origins, living in the Łódź region of central Poland. Informed consent was obtained from patients and controls.

Socio-demographic data, health-related information and cigarette smoking status were obtained from each participant. In all cases, surveys were performed to complete the tumor registry database. The database catalog was queried every 6 months and all histopathologically confirmed incidents of sinonasal inverted papilloma cases reported within 6 months of diagnosis preceding the recruitment were identified. Smoking status was categorized into “current”, “former”, and “never” based on self-reported usage. Individuals who reported smoking at least 100 cigarettes in their lifetime and who, at the time of survey, smoked either every day or some days were classified as a current smoker. Individuals who reported smoking at least 100 cigarettes in their lifetime and who had not been smoking for at least 3 months were defined as a former smoker. Participants who reported never having smoked 100 cigarettes were defined as a never smoker. A positive family history of head and neck region neoplasms was defined as being self-reported in at least one first-degree relative with known neoplastic disease.

### Histological classification and morphological features

All tissue samples were fixed in 10 % neutral buffered formalin, embedded in paraffin, and routinely processed for histological examination. Archival paraffin-embedded tissue samples were used for the histological classification of tumors. At this point in the study, all formalin-fixed, paraffin-embedded (FFPE) slides were carried out in a single reference laboratory and evaluated by two independent pathologists without prior knowledge of patient clinical data. When differences in opinion occurred between the two investigators, agreement was reached by subsequent careful discussion. The inverted papilloma tissues used in this study in each case were selected individually from several FFPE tissue sections which had been estimated pathologically. H&E-stained sections provided a histological confirmation of IP. Sinonasal inverted papillomas were classified with the Krouse staging system [28]. The IP were typed histopathologically according to the IARC WHO classification of head and neck tumors [29]. Morphological estimation was performed on H&E-stained sections in at least five different regions of the invasive, peripheral part of the tumor (×200 magnification). The histological evaluation considered the histological type (exophytic and endophytic growth), the presence of dysplasia (mild or severe), bone destruction, anatomical area occupation and any association with malignancy.

### Immunohistochemistry

Paraffin-embedded tissue sections were mounted onto SuperFrost slides, deparaffinized, then treated in a microwave oven in a solution of target retrieval solution (TRS, pH 6.0, Dako) for 30 min (2 × 6 min 360 W, 2 × 5 180 W, 2 × 4 min 90 W) and transferred to distilled water. Endogenous peroxidase activity was blocked by 0.3 % hydrogen peroxide in distilled water for 30 min, and then sections were rinsed with Tris-buffered saline (TBS, Dako, Denmark) and incubated 30 min with primary mouse monoclonal antibodies against: Ki67 (Dako; clone MIB1, dilution 1:50). Immunoreactive proteins were visualized using EnVision-HRP kit (Dako, Carpinteria, CA, USA) according to the instructions of the manufacturer. Visualization was performed by incubation the sections in a solution of 3,3′-diaminobenzidine (DakoCytomation, Denmark). After washing, the sections were counter-stained with hematoxylin and coverslipped. For each antibody and for each sample, negative controls were processed. Negative controls were carried out by incubation in the absence of the primary antibody and always yielded negative results. All immunohistochemical analyses were carried out in a single reference laboratory and evaluated by light microscopy blindly and independently by two pathologists. Ki67 staining was performed routinely in all cases of dysplasia and/or in those cases in which the histological evaluation of H&E-stained sections aroused any suspicion of increased proliferation of tumor cells. Ki67 was scored as the percentage of nuclei-stained cells out of all tumor cells in the front of the neoplasm regardless of the intensity in ×400 high-power field, 500 to 1000 tumor cells were counted in each case. We classify IHC Ki67 expression into two categories according to the score of Ki67: low (<14 % Ki67-positive cells) and high (≥14 % Ki67-positive cells).

### Sample collection and DNA and extraction from FFPE

The tissue specimens from sinonasal inverted papilloma collected in the operation room were prepared and evaluated by an experienced pathologist as mentioned above. Sections were deparaffinized by two rinses in xylene and ethanol. After deparaffinization, samples were rehydrated. The tissue was collected by centrifugation. After the final wash, alcohol was aspirated and the tissue pellets were resuspended in digestion buffer (10 mM NaCl, 500 mM Tris-HCl, pH 8.0, 25 mM EDTA, 1 % SDS) and 1 mg/ml proteinase K was added. Sections were incubated at 50 °C overnight. Prior to deoxyribonucleic acid (DNA) purification, proteinase K was inactivated by incubation at 97 °C for 10 min. The digested samples were extracted using TRI Reagent (Sigma Aldrich Co., USA) according to the manufacturer’s protocol. Venous blood samples were obtained from each volunteer (10 ml) and transferred to test tubes containing EDTA as an anticoagulant. After blood collection, the tube was inverted 8–10 times and placed in a refrigerator at −20 °C. Blood samples obtained from the participants were stored at −20 °C within 2 h of removal. DNA was isolated by standard method using proteinase K digestion, phenol chloroform extraction and ethanol precipitation. The investigations were performed with the approval of the Bioethical Commission of the Medical University of Łódź and the National Science Council, Poland (approval No RNN/60/13/KE).

Genotyping for the rs28366003, rs1610216, and rs10636 allelic variants was performed as described previously [18, 19].

### Statistical data analysis

Genotype distributions were evaluated for agreement with Hardy-Weinberg equilibrium by the Chi-square test (*χ* ^2^). All genotype distributions of MT2A fit the Hardy-Weinberg equilibrium. Unconditional multiple logistic regression models were used to calculate odds ratios (ORs) and 95 % confidence intervals (CIs) for the association of genotype with inverted papilloma risk. Genotype data was analyzed with the homozygote of the common allele as the reference group. Variants of homozygotes and heterozygotes were combined to evaluate the dominant effect. For each SNP, trend tests were conducted by assigning the values 1, 2, and 3 to homozygous wild type, heterozygous, and homozygous variant genotypes, respectively, and by adding these scores as a continuous variable in logistic regression model.

The haplotype effects of the polymorphisms on inverted papilloma risk were analyzed using Chaplin 1.2 (genetics.emory.edu) and THESIAS software (www.genecanvas.org). All haplotypes were examined simultaneously in regression models with the most common haplotype as the reference. Haplotypes were evaluated for association with IP in unadjusted and adjusted logistic regression models as for the individual SNPs. All multivariate models were adjusted for age, gender, family history, and smoking status. Reported *p* values were two sided. Probabilities were considered significant whenever the *p* value was lower than 0.05. All analyses were completed using STATA software (version 11.0 Stata-Corp., Texas, USA).

## Results

**Fig. 1:**
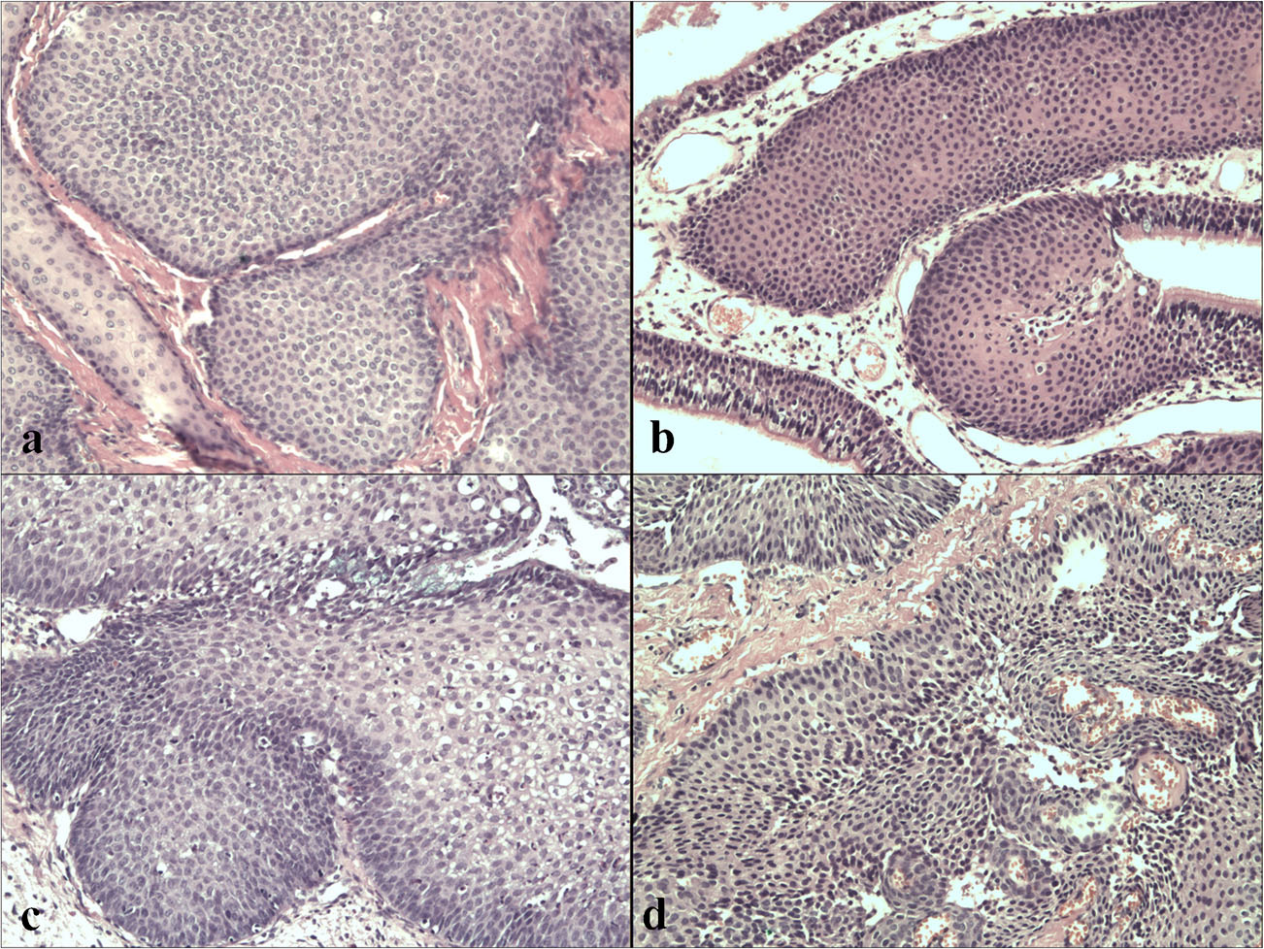
Inverted papilloma without dysplasia (H&E, ×200 magnification) (**a**), inverted papilloma without dysplasia and prominent stromal edema (H&E, ×200 magnification) (**b**), inverted papilloma with mild dysplasia and signs of viral infection (H&E, ×200 magnification) (**c**), inverted papilloma with severe dysplasia (H&E, ×200 magnification) (**d**)

**Fig. 2:**
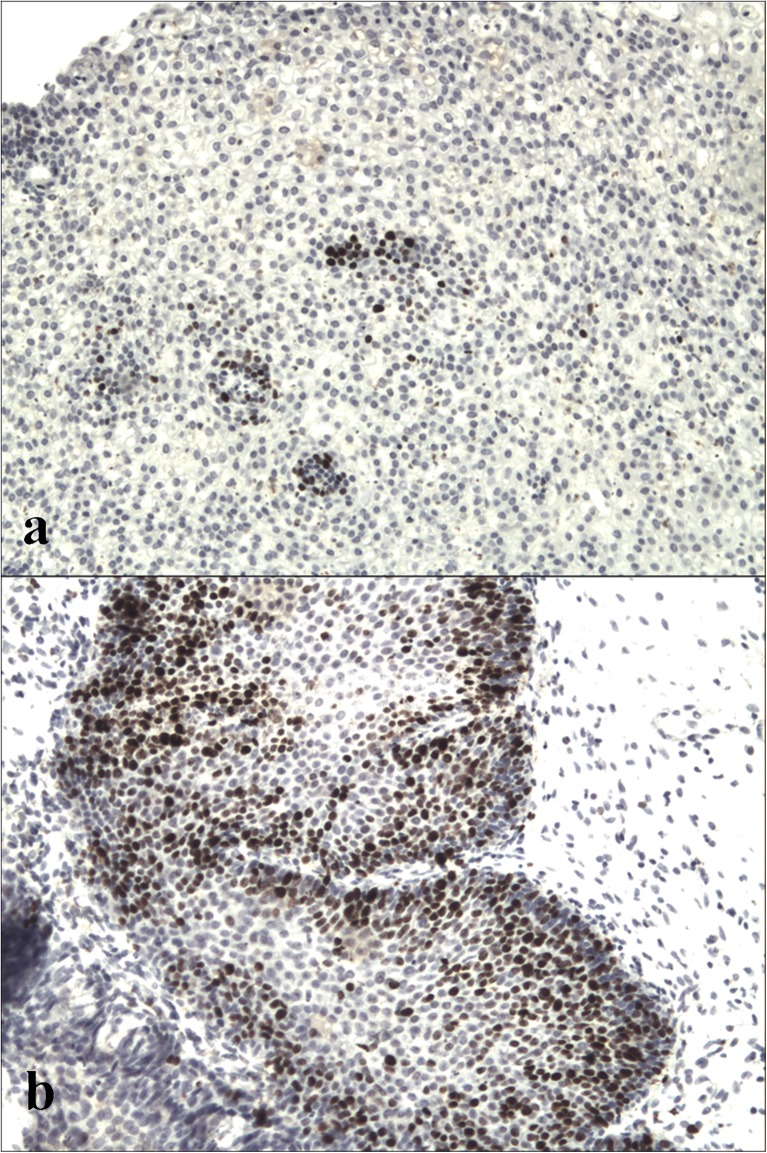
Ki67 immunohistochemistry (IHC): the low immunoexpression of Ki67 in inverted papilloma without dysplasia (×100 magnification) (**a**), the high immunoexpression of Ki67 in inverted papilloma with severe dysplasia (×100 magnification) (**b**)

The distribution of socio-demographic features of the study subjects is shown in Table 1. All of the studied individuals, patients and controls were Caucasians and constituted a homogenous population from the same ethnic and geographical origins. On average, the cases with identified histopathologically sinonasal inverted Schneiderian papilloma (IP) were slightly younger than controls (59.14 years ± 12.66 vs. 64.17 years ± 8.28 for controls) and more often qualified as current smokers (54.6 vs. 33.7 % for controls). A positive family history of benign or malignant head and neck region tumors in at least one first-degree relative was mildly significant compared to controls (3.1 vs. 4.1 % for controls). The clinicopathological parameters of the inverted papillomas studied are presented in Table 2. In the histological examination tumoric lesions in most cases were typed as endophytic (91.5 %) according to the IARC WHO classification. IP cases more likely to have diffuse tumor growth, as defined by a simultaneous invasion of more anatomical sites (53.1 %) and they appeared to dominate by pT2 (58.5 %) tumors according to Krouse staging with maxillary or ethmoid anterior sinuses occupation. Mild or severe dysplasia in tumor stroma and invasive bone destruction were observed in 32.3 and 15.4 % tumors, respectively. Typical histopathological features of the inverted Schneiderian papilloma showing endophytic growth of pseudostratified ciliated epithelium forming cystic spaces with and without dysplastic areas are shown in Fig. 1a–d. The locoregional recurrences were confirmed in almost 14 % cases. Of the 51 cases (42 cases of dysplasia and 9 IP cases in which the histological evaluation of H&E-stained sections disclosed an increased proliferation of tumor cells), 46 (35.4 %) tumors have high IHC Ki67 index, and only 5 (3.8 %) cases were Ki67 low-stained. The representative images of low and high Ki67 immunoexpression in tissues of inverted papilloma are shown in Fig. 2a, b.

**Table 1:** Socio-demographic of inverted papilloma cases and controls

	Cases *n* (%)	Controls *n* (%)	*p* ^a^
Sample size	*n* = 130	*n* = 418	
Observation period, months	59.37 ± 37.63	84.56 ± 35.44	
Age, years	59.14 ± 12.66	64.17 ± 8.28	
<50 years	49 (37.7)	190 (54.5)	
≥50 years	81 (62.3)	228 (45.5)	0.11
Gender			
Male	89 (68.5)	314 (75.1)	0.13
Female	41 (31.5)	104 (24.9)	
Family history of H&N tumors*			
Yes	4 (3.1)	17 (4.1)	0.61
No	126 (96.1)	401 (95.9)	
Smoking status^b^			
Smokers (current)	71 (54.6)	141 (33.7)	<0.001
Non-smokers	59 (45.4)	277 (66.3)	
Former	12 (9.2)	155 (37.1)	
Never	47 (36.2)	122 (29.2)	

^a^The chi-square (*χ* ^2^) test

^b^Smoking was grouped into “current”, “former” and “never” based on self-reported usage. Participants who reported smoking at least 100 cigarettes in their lifetime and who, at the time of survey, smoked either every day or some days were defined as a current smoker. Participants who reported smoking at least 100 cigarettes in their lifetime and who had not been smoking for at least 3 months were defined as a former smoker. Participants who reported never having smoked 100 cigarettes were defined as a never smoker

*H&N tumors* benign or malignant tumors of head and neck region

**Table 2:** Clinicopathological characteristics of inverted papilloma cases

	Variable	Cases *n* (%)
Symptoms	Nasal obstruction	64 (49.2)
	Rhinorhoea	71 (54.6)
	Feeling of pressure	24 (18.5)
	Impaired sense of smell	50 (38.5)
	Pain	20 (15.4)
	Epistaxis	9 (10.8)
	Diploplia	3 (2.3)
	None	8 (6.2)
Stage (Krouse classification)^a^	pT1	40 (30.7)
	pT2	76 (58.5)
	pT3	13 (10.0)
	pT4 (*squamous cell carcinoma*)	1 (0.8)
Histological type^b^	Endophytic growth	119 (91.5)
	Exophytic growth	11 (8.5)
	Cylindrical cell papilloma	0 (0.0)
Anatomical area occupied^c^	Nasal cavity	97 (74.6)
	Maxillary sinuses	70 (53.8)
	Ethmoid anterior sinuses	43 (33.1)
	Ethmoid posterior sinuses	15 (11.5)
	Frontal sinuses	8 (6.1)
	Sphenoid sinuses	6 (4.6)
Anatomical sites occupied	1 region	61 (46.9)
	More regions	69 (53.1)
IP with dysplasia (mild or severe)	No	88 (67.7)
	Yes	42 (32.3)
Ki67 IHC staining^d^	Low	5 (3.8)
	High	46 (35.4)
Bone destruction	No	110 (84.6)
	Yes	20 (15.4)
Recurrences of IP	No	112 (86.2)
	Yes	18 (13.8)
IP associated with malignancy	No	129 (99.2)
	Yes	1 (0.8)

^a^Krouse staging system [28]

^b^The IARC WHO classification of head and neck tumors [29]

^c^In each IP case, one or more anatomical sites may be occupied simultaneously

^d^Low (<14 % Ki67-positive cells); high (≥14 % Ki67-positive cells)

Genotype and allele distributions in the *MT2A* gene in 130 genetically unrelated inverted papilloma patients and 418 controls are summarized in Table 3. All genotype and allele frequencies (−5 A/G, −209 A/G and +838 C/G) were confirmed as compatible with Hardy-Weinberg equilibrium among the cases and controls, respectively (all *p* > 0.05). As a result, only the −5 A/G (rs28366003) SNP in the *MT2A* gene was significantly related to Schneiderian papilloma in this population-based case-control study. The frequency of A allele carriage was 99.2 and 100 % in cases and controls, respectively. The G allele carriage was detected in 24.6 % of IP and in 4.1 % of the non-IP samples. The respective genotype frequencies in the promoter region at locus −5 (rs28366003) were found as homozygote typical (A/A), heterozygote (A/G) and homozygote atypical (G/G) in 75.4, 23.8, and 0.8 % for cases, and 95.9, 4.1, and 0 % for controls. Likewise, genotype distribution with regard to age, gender and smoking status was found to be very similar. *MT2A* gene SNP in relation to age, gender, and smoking status in cases and controls are shown in Table 4. A significant interaction with gender for rs28366003 was observed in cases (*p* = 0.006). This suggests an influence of sex on the association of *MT2A* gene SNP with IP. It should be also emphasized that IP individuals with confirmed recurrences in follow-up in most cases appeared to have heterozygosity (A/G) at locus −5 (rs28366003) (55.6 %). The homozygote typical (A/A) constituted only 44.4 % of all these cases. Mild or severe dysplasia was observed in 47.6 and 52.4 % carriers of the G and A allele, respectively. All cases with low IHC Ki67 index were individuals with A/A genotype. Bone destruction was confirmed in 55 % of individuals with one copy of the risk allele (G) at locus −5 (rs28366003) MT2A and in 45 % of typical homozygotes. Malignant transformation into squamous cell carcinoma also concerned individuals with an A/G genotype.

**Table 3:** Associations between *MT2A* SNPs and inverted papilloma risk

SNP genotype	Cases (%)/controls (%)	OR (95 % CI)^a^	*p*	OR (95 % CI)^b^	*p*
rs28366003					
AA	98 (75.4)/401 (95.9)	1.00 (reference)		1.00 (reference)	
AG	31 (23.8)/17 (4.1)	7.46 (3.84–14.47)	<0.001	7.43 (3.91–14.46)	<0.001
GG	1 (0.8)/0 (0.0)	–			
*p* trend^c^	<0.0001				
AG or GG vs. AA^d^	32 (24.6)/17 (4.1)	7.70 (3.97–14.91)	<0.001	7.71 (4.01–14.91)	<0.001
AG or AA vs. GG^e^	129 (99.2)/418 (100.0)	–		–	
rs1610216					
AA	96 (73.8)/309 (73.9)	1.00 (reference)		1.00 (reference)	
AG	33 (25.4)/106 (25.3)	1.00 (0.64–1.58)	0.99	1.01 (0.66–1.58)	0.99
GG	1 (0.8)/3 (0.8)	1.07 (0.11–10.46)	0.95	1.07 (0.10–10.44)	0.94
*p* trend^c^	0.97				
AG or GG vs. AA^d^	34 (26.1)/109 (26.1)	1.00 (0.64–1.57)	0.99	0.99 (0.61–1.56)	0.99
AG or AA vs. GG^e^	129 (99.2)/415 (99.3)	1.07 (0.11–10.42)	0.95	1.07 (0.13–10.42)	0.95
rs10636					
GG	58 (44.6)/213 (50.9)	1.00 (reference)		1.00 (reference)	
GC	56 (43.1)/172 (41.2)	1.19 (0.78–1.82)	0.40	1.23 (0.72–1.82)	0.40
CC	16 (12.3)/33 (7.90)	1.78 (0.91–3.47)	0.09	1.78 (0.94–3.47)	0.09
*p* trend^c^	0.1				
GC or CC vs. GG^d^	72 (55.4)/205 (49.0)	1.29 (0.87–1.92)	0.21	1.30 (0.87–1.95)	0.22
GC or GG vs. CC^e^	114 (87.7)/385 (92.1)	1.64 (0.87–3.09)	0.12	1.65 (0.83–3.10)	0.13

^a^Crude

^b^Adjusted for age, gender, family history and smoking status

^c^Testing additive genetic model (Cochran-Armitage test for trend)

^d^Testing dominant genetic model

^e^Testing recessive genetic model

**Table 4:** *MT2A* gene −5 A/G rs28366003 polymorphism in relation to age, gender and smoking status in an inverted papilloma (cases) and non-papilloma cases (controls)

Variable		Genotype *n* (%)			*p* ^a^
		A/A	A/G	G/G	
IP—cases					
Age	<50 years (*n* = 49)	40 (30.8)	8 (6.1)	1 (0.8)	0.42
	≥50 years (*n* = 81)	58 (44.6)	23 (17.7)	0 (0.0)	
	<50 years (years ± SD)	47.9 ± 15.8	46.8 ± 12.34	47	-
	≥50 years (years ± SD)	62.1 ± 13.9	52.0 ± 16.5	-	
Gender	Male (*n* = 89)	59 (45.4)	29 (22.3)	1 (0.8)	0.006
	Female (*n* = 41)	39 (30.0)	2 (1.5)	0 (0.0)	
Smoking status	Smokers (*n* = 71)	51 (39.2)	19 (14.6)	1 (0.9)	0.77
	Non-smokers (*n* = 59)	47 (36.1)	12 (9.2)	0 (0.0)	
non-IP—controls					
Age	<50 years (*n* = 190)	185 (44.2)	5 (1.2)	0 (0.0)	0.17
	≥50 years (*n* = 228)	216 (51.7)	12 (2.9)	0 (0.0)	
	<50 years (years ± SD)	53.1 ± 14.6	46.2 ± 11.4	–	–
	≥50 years (years ± SD)	67.3 ± 12.5	51.1 ± 12.1	–	
Gender	Male (*n* = 314)	305 (73.0)	9 (2.1)	0 (0.0)	0.03
	Female (*n* = 104)	96 (23.0)	8 (1.9)	0 (0.0)	
Smoking status	Smokers (*n* = 141)	128 (30.6)	13 (3.1)	0 (0.0)	0.0001
	Non-smokers (*n* = 277)	273 (65.3)	4 (1.0)	0 (0.0)	

^a^The chi-square (*χ*2) test

Our findings reinforce the association of MT2A (rs28366003) with the risk of Schneiderian papilloma. In the study population, the pathogenic link between the rs1610216 and rs10636 variants and this type of tumor was excluded. The genetic association analysis revealed that the presence of A/G allele at *MT2A* gene were at higher risk with an approximately 7.4-fold increase for the development of sinonasal inverted papilloma (odds ratio [OR] = 7.43; 95 % confidence interval [CI], 3.91–14.46; *p* < 0.001). Moreover, confirmation of one copy of the risk allele (G) at locus −5 (rs28366003) of the core promoter region of *MT2A* gene conferred an estimated increase in Schneiderian papilloma of almost 7.7-fold in the model adjusted for age, gender, family history and smoking status (OR = 7.71; 95 % CI, 4.01–14.91, *p*_dominant_ < 0.001). Individuals with A/A genotype have a protective effect against IP development. Four MT2A haplotypes were shown to have a population frequency of at least 5 %. Interestingly, of the four coding variants found solely in IP patients, a three-locus haplotype (G-A-G) including the rs28366003 SNP variant allele of the *MT2A* gene was found to be significantly associated with a 16 % increased risk of inverted papilloma (OR = 1.16; 95 % CI, 0.74–1.94, *p*_global_ = 0.02). The association between MT2A haplotypes and statistical analysis results of Schneiderian papilloma risk are shown in Table 5.

**Table 5:** Associations between *MT2A* haplotypes and inverted papilloma risk

Haplotypes (SNP1–SNP3)	Cases (%)/controls (%)	OR (95 % CI)^a^	OR (95 % CI)^b^
A-A-G	53 (40.8)/133 (31.8)	1.00 (reference)	1.00 (reference)
G-A-G	41 (31.5)/87 (20.8)	1.18 (0.72–1.93)	1.16 (0.74–1.94)
A-A-C	21 (16.1)/104 (24.9)	0.51 (0.28–0.89)	0.49 (0.27–0.89)
A-G-C	15 (11.6)/94 (22.5)	0.40 (0.21–0.76)	0.40 (0.24–0.75)

^a^Crude

^b^Adjusted for age, gender, family history, and smoking status

Since a significant association was confirmed between −5 A/G SNP and the risk of inverted papilloma, it was investigated whether genetic variation identified in the core promoter region can affect tumor behavior, as defined by morphological evaluation. The SNP at locus −5 (rs28366003) was also found to be associated with IP phenotype in a Polish population. The heterozygotes of the major allele and atypical homozygotes appeared to have higher Krouse stage (pT1 vs. pT2–4) (OR = 19.32; 95 % CI, 2.30–173.53; *p* < 0.0001), more diffuse tumor growth, as defined by a simultaneous occupation of more anatomical sites (OR = 4.58; 95 % CI, 1.70–12.11; *p* = 0.0008) and bone destruction (OR = 4.13; 95 % CI, 1.50–11.60; *p* = 0.003). A similar significant association was determined for the risk allele carriage with regard to higher incidence of tumor recurrences in follow-up (OR = 5.11; 95 % CI, 1.68–15.20; *p* = 0.001). Confirmation of one copy of the risk allele (G) in the core promoter region of *MT2A* gene conferred an estimated five-fold increase in local relapse of neoplastic process. The associations between clinicopathological characteristics and papilloma inverted risk are shown in Table 6.

**Table 6:** Associations between clinicopathological characteristics and papilloma inverted risk

Variable	SNP rs28366003		OR (95 % CI)^a^	*p*	OR (95 % CI)^b^	*p*
	AA *n* (%)	AG or GG *n* (%)				
Stage (Krouse system) pT1 vs. pT2–T4	39 (30.0)/59 (45.4)	1 (0.8)/31 (23.8)	20.49 (2.34–179.64)	<0.0001	19.32 (2.30–173.53)	<0.0001
Histological type exo vs. endophytic	11 (8.5)/87 (66.9)	2 (1.5)/30 (23.1)	1.89 (0.39–9.14)	0.41	1.87 (0.40–9.14)	0.42
Anatomical area^c^ NS/M/EA vs. EP/F/S	81 (62.3)/17 (13.1)	22 (16.9)/10 (7.7)	2.16 (0.86–5.47)	0.09	2.15 (0.86–5.39)	0.08
Anatomical sites 1 region vs. more	55 (42.3)/43 (33.1)	7 (5.4)/25 (19.2)	4.57 (1.72–12.11)	0.0008	4.58 (1.70–12.11)	0.0008
Dysplasia no vs. yes (mild/severe)	76 (58.5)/22 (16.9)	12 (9.2)/20 (15.4)	2.13 (0.92–4.90)	0.07	2.14 (0.97–4.91)	0.07
Bone destruction no vs. yes	85 (65.4)/9 (6.9)	25 (19.2)/11 (8.5)	4.15 (1.49–11.60)	0.003	4.13 (1.50–11.60)	0.003
Recurrences no vs. yes	90 (69.2)/8 (6.2)	22 (16.9)/10 (7.7)	5.11 (1.71–15.21)	0.001	5.11 (1.68–15.20)	0.001

^a^Crude, AA vs. AG or GG *MT2A* SNP rs28366003 (the homozygote of the common allele were estimated as the reference group)

^b^Adjusted for age, gender, family history and smoking status

^c^In NS/M/EA cases only nasal cavity, maxillary sinuses and/or ethmoid anterior sinuses were occupied; In EP/F/S cases besides nasal cavity, maxillary sinuses and/or ethmoid anterior sinuses the other anatomical sites such as ethmoid posterior sinuses, frontal sinuses and/or sphenoid sinuses were occupied simultaneously (*NC* nasal cavity, *M* maxillary sinuses, *EA* ethmoid anterior sinuses, *EP* ethmoid posterior sinuses, *F* frontal sinuses, *S* sphenoid sinuses)

## Discussion

The present work evaluates the effect of genetic polymorphisms in the MT2A core promoter region on the risk and tumor behavior of sinonasal Schneiderian papilloma in a Polish population. It should be emphasized that, at the time of writing, no publication has yet analyzed the SNPs of MT2A in relation to human inverted papilloma susceptibility. Moreover, this is also the first study to investigate the expression of MT2A isoforms in IP biopsy tissue and to document the relationship between MT2A SNPs and aggressive phenotype of this type of head and neck tumor. Therefore, our findings may only be compared with those of studies carried out for other types of head and neck cancers or human neoplasms in other localizations. It should also be noted that, to our knowledge, the material studied constitutes the largest homogeneous group of these benign, locally aggressive head and neck tumors which share both the same tissue origin and histological type according to the IARC WHO classification. This is an additional value of this research, when the difficulties in obtaining such rare pathological material collected over many years are taken into consideration. Because of these constraints, an overwhelming proportion of inverted papilloma research has used only paraffin-embedded archival material, often histologically heterogenous and thus limited in number, with fewer than fifty cases being included in most studies. The limitations described doubtlessly have an impact on the final results and the data interpretation in terms of the mechanisms of the tumorigenesis of this neoplastic lesion. Hence, greater knowledge of the SNPs of MT2A and their relationship with human inverted papilloma tumorigenesis and behavior is needed to elucidate the biological functions of metallothioneins in this neoplastic process, and to determine their possible clinical significance as biomarker of the dynamics of tumor growth, invasiveness, and prognosis. Three analyzed SNPs that could potentially affect *MT2A* gene expression were selected on the basis of a literature review.

In recent years, the possible role of various metallothionein isoforms and polymorphisms on physiological and pathological processes has been documented [6, 10, 30]. Since the expression of MTs can be affected by genetic variation in the core promoter region and diverse frequency of the essential alleles, the presence of SNPs may lead to reduced cellular activity of metallothioneins and affect abnormal intracellular pathways of cell proliferation, apoptosis, and response to oxidative stress, thus potentially influencing the susceptibility of the individual to various types of tumors [6, 8, 20–22, 31–33]. Unfortunately, among the several previous reports on the rs28366003, rs1610216, and rs10636 SNPs in the *MT2A* gene, only single studies related to the neoplastic process report the frequency of MT isoforms within different ethnic populations [20–23, 34–38].

Our study documents for the first time the genetic association between the occurrence of −5 A/G SNP in the core promoter region of the *MT2A* gene and the risk of sinonasal inverted papilloma, as well as the dynamics of tumor growth according to Krouse staging, a precise, multifactorial histological analysis of biopsy material and incidence of recurrences. The harvested data indicates that a positive relationship exists between (rs28366003) SNP and both higher susceptibility and increased invasiveness of Schneiderian papilloma in Polish inhabitants.

As a result, the genotype frequencies in the core promoter region at locus −5 A/G (rs28366003) were found to be homozygote typical (A/A) in 75.4 %, heterozygote (A/G) in 23.8 % and homozygote atypical (G/G) in 0.8 % individuals with sinonasal inverted papilloma. Our recent study has also reported a similar prevalence of A by G replacement at position −5 in the core region of the MT2A promoter for homozygous common allele carriers as well as for heterozygosity and homozygosity for the G variant in the studied IP group [39]. The resulting data resemble the findings in some ethnic populations such as those from Japan, Turkey, and America [23, 36–38]. For example, Kita et al. [23] report that an incidence of the conversion A → G in the core promoter region of the *MT2A* gene near the TATA box was 18 % for Japanese people, with the carriage of different genotypes being 82, 17, and 0.9 % for A/A, A/G, and G/G, respectively. Similar results were detected by Kayaalti and Söylemezoğlu [37] within a Turkish population, wherein the genotype frequencies of −5 A/G SNP were observed as homozygote typical in 87 %, heterozygote in 12.3 %, and homozygote atypical in 0.7 % individuals. In another study, the same researchers observe a high dependence of MT2A core promoter gene polymorphisms with aging and conclude that absence of a risk allele variant (G) may be associated with better survival [36]. Similarly, McElroy et al. [38] demonstrated the allele G was not frequent genetic variation at rs28366003 in *MT2A* gene in black and white female volunteers in the Midwestern United States. In this population, the prevalence of the minor allele was found to be 6.4 % for white and 1.1 % for black individuals.

The data concerning MT2A genotype frequencies identified in the different neoplastic processes were also found to be very similar to our results: the most frequent genotype was A/A, while heterozygosity and homozygosity for the risk G variant was less so [20–22]. The genotype distributions of MT2A (rs28366003) SNP in prostate cancer and breast cancer described by Krześlak et al. [20, 21] confirm those given in the present study and were found to be A/A in 76 %, A/G in 21.1 %, G/G in 2.9 % and A/A in 87.1 %, A/G in 12.3 %, G/G in 0.6 % in prostate and breast cancer cases, respectively.

The present study documents the contribution of the −5 A/G single-nucleotide polymorphism in the *MT2A* gene toward the risk of developing inverted papilloma in a Polish population. It was also determined that confirmation of one copy of the risk allele (G) at locus −5 (rs28366003) of the core promoter region of *MT2A* gene conferred 7.7-fold increase in Schneiderian papilloma risk in a model adjusted for age, gender, family history, and smoking status.

Unfortunately, as a literature survey revealed no publication describing a genetic implication of MT2A SNPs in the pathogenesis of head and neck tumors, the harvested results can only be compared to our previous reports in laryngeal cancer [18, 19]. The results indicate that the presence of an SNP in the core promoter region at locus −5 A/G (rs28366003) in the *MT2A* gene contributes to a higher risk of malignant head and neck tumors.

It should be also added that only a few studies found in the literature concern metallothionein genetic variations in relation to pathological mechanisms, as well as to the development, tumor behavior, and progression of other neoplastic lesions [20–22, 31–33]. The studies indicate that MT2A core promoter region genetic polymorphism and in particular confirmation although of one copy of the risk allele at rs28366003 in the *MT2A* gene may have an impact on cancer risk of various origins. In this context, our findings for sinonasal inverted papilloma resemble the findings in other cancers. Aberrant MT expression due to altered transcription at the core promoter region of the *MT2A* gene, as a reason of enhanced sensitivity for the carcinogenic heavy metals and metallic agents and deregulation of cell proliferation and inhibition of apoptosis, have been discussed [5, 20–22, 32, 33]. For example, Forma et al. [22] demonstrated almost 1.3- and 4.3-fold greater risks of primary prostate cancer associated with SNP in MT2A (rs28366003) linked to heterozygosity and homozygosity for the risk G allele, respectively, in the model adjusted for age. A similar result was reported by Krześlak et al. [21] for the same type of cancer in which a 2.6-fold increase in risk in heterozygous G allele carriers compared to individuals who were homozygous for allele A was established. Moreover, it was demonstrated that enhanced susceptibility to neoplasm was related to aberrant expression of MT2A and Cd, Zn, Cu, and Pb content in biopsy neoplastic tissue. In another study, the same researchers also observed an increase of almost 90 % in the risk of ductal breast cancer after adjustment for inter alia age, family history, smoking status, menarche and menopausal status in subjects having one copy of the risk G allele in MT2A promoter region as well as an increase of 50 % in risk of cancer when MT2A haplotypes G-A-G was taken into account. An association between other *MT2A* SNPs and susceptibility to tumorigenesis was also detected, confirming several previous reports. For instance, Seibold et al. [32, 33] documented a significant modification of post-menopausal breast cancer risk per specific alleles of six polymorphisms, including SNP in MT2A (rs1580833) and its association with the overall mortality of the subjects. Regardless of different types of cancers discussed in the literature, the findings are in line with other research suggesting potential role of MT2A SNP in determining the risk of neoplastic process.

Since the conversion of nucleotides A to G in the core promoter region of *MT2A* gene may affect the MT gene transcription induced by heavy metals and metallic agents and consequently lead to higher sensitivity to metal toxicity, the risk of tumors of various origin due to SNP has been evaluated in certain tumors [5, 20–23]. The direct functional connections and an understanding of the specific mechanisms through which the SNP in the MT2A gene regulate toxic metal concentrations as potential carcinogenic factors and influence tumor cell dynamics and pathology have been reported in only a few publications and remain elusive [5, 8, 20, 33–35, 40–42]. The study by Kayaalti et al. [34] provides evidence implicating MT2A SNP polymorphisms on heavy metal expression in the blood samples taken from a Turkish population. A confirmation of homozygosity for the risk allele variant (G) at locus −5 (rs28366003) in *MT2A* gene determined higher Cd and Pb concentration compared with A/A and A/G genotypes, as well as higher sensitivity for heavy metal toxicity. However, in contrast to the present study, the same authors report that certain genetic variations such as heterozygosity and atypical homozygosity influence higher accumulation of Cd in autopsy kidney tissues, but no association was found between −5 A/G SNP and both Zn and Cu levels in the renal cortex [35]. Also, Tekin et al. [43] observed the implication of A/G genotype carriage in the MT2A promoter region on the increased Pb concentration in blood samples and placental tissue in pregnant women and the risk of low-level cord blood lead variation in their newborns. The harvested data resemble our recent findings in inverted papilloma, in which a significant connection was observed between rs28366003 and a considerably high accumulation of both Cd and Cu in biopsy tissue [39]. These changes in toxic metal levels in neoplastic tissue remain in line with our previous studies on laryngeal cancer, which note that −5 A/G SNP in the *MT2A* gene may be related to Cd, Zn, and Cu content [18].

Our study also documents the relationship between the SNPs in the *MT2A* gene and the dynamics of tumor growth, according to a thorough multifactorial histological evaluation of inverted papillomas. The current data confirms that the carriage of the risk allele (G) was associated with increased tumor invasiveness according to Krouse grading, and a higher incidence of recurrences when compared with typical homozygous patients. This data is also confirmed by our previous study of laryngeal cancers, which notes that −5 (rs28366003) of the core promoter region of *MT2A* gene was found to exert issue impact on malignant tumor behavior [19].

Unfortunately, a literature search indicates that no studies have yet been performed on the relationship between SNPs at rs28366003 MT2A and phenotype of aggressiveness in tumors of the head and neck region. However, some studies have linked MT isoform function to neoplastic cell dynamics and tumor pathology in other neoplasms, yet findings remain limited and often divergent, especially regarding the impact of their specific isoforms on tumor invasion and prediction of patient outcome [5, 10, 20–23]. Most researchers have confirmed increased expression of MT1 and MT2 and a positive association of these isoforms with standard clinical parameters such as higher pTNM classification, higher stage, lower histological differentiation as well as chemotherapy and radiation resistance and shorter survival time. However, MT expression does not affect the clinical disease parameters and mortality in other types of tumors, i.e., hepatocellular, gastric, colorectal, central nervous system, and thyroid cancers [10, 25, 44].

Nevertheless, contrary results for MT expression related to tumor behavior, chemo-, and radiotherapy response and prognosis in head and neck neoplasms have also been reported in a few available studies [10, 26, 27, 45]. For instance, Pedersen et al. [10] report increased expression of MT1 and MT2 isoforms in human head and neck primary cancers of the nasopharynx, salivary gland, thyroid, and larynx. Also, Theocharis et al. [26] determined the impact of MT cellular distribution in either the tumor itself, or adjacent epithelial mucosa tissue on the development and progression of mobile tongue cancer. The authors note that the occurrence of positive MT expression is frequently characteristic of tumors with lower histological grade, vascular invasion, deep infiltration, and the existence of lymph node metastases. Similarly, Gumulec et al. [14] estimate a positive association of MT immunoexpression with advanced tumor grade and shorter patient survival in head and neck malignant tumors. By contrast, other researchers have not confirmed that MT expression corresponds to clinicomorphological parameters in tumors of the head and neck region [27, 45]. For example, Pastuszewski et al. [27] document the opposite findings in laryngeal carcinomas. In this case, MT expression was found to be not significantly related to the tumor grade, stage or clinical outcome parameters. The results described by Dutsch-Wicherek et al. [45] were almost the same as those given in the mentioned study. MT staining in tumor infiltration was lower than in peri-tumor stroma tissue and has been found not to be associated with nodal metastases in head and neck cancer and breast adenocarcinoma population. The researchers suggest that MT expression may be due to a protective reaction of healthy noncancerous tissue to the tumor antigens.

In addition, a literature review reveals a wide range of findings concerning MT expression also identified in other types of neoplastic lesion. Most of the data indicate that MT2A transcripts or, most often, protein expression in tumors may be involved in determining aggressive tumor behavior and increased patient mortality, however, many demonstrate equivocal results and divergent conclusions [16, 17, 25, 44, 46–48]. For example, Werynska et al. [48] report enhanced MT2A isoform expression in a studied population of non-small cell lung cancer cases, and identify a positive association between MT immunostaining and primary tumor size, grade of malignancy and poor prognosis. Similarly, Kobierzycki et al. [46] observe MT1/MT2 cytoplasmic overexpression in neoplastic tissues in ovarian cancer and conclude these proteins can be predictive markers for advanced stage. Also, Habel et al. [16] indicate that higher MT2A level may also correspond to lower mortality rate and has an effect on chemoresistance to cytotoxic drugs in patients treated with osteosarcoma. A similar result was reported by Gumulec et al. [25] for prostate cancer model. In this study, upregulation of MT2A at the protein level was established to be involved in CDDP resistance. However, Gansukh et al. [44] demonstrate no significant correlation between MT expression and cisplatin sensitivity in non-small cellular lung cancer. Liang et al. [47] also suggest that the downregulation of MT2A gene level, rather than increased MT expression, may play the key role in lung carcinogenesis. Similarly, Pan et al. [17] suggest that MT2A exerts tumor suppressive activity and observe that decreased MT2A expression in cell lines and primary tumors correlates with advanced clinical stage and poor prognosis in gastric cancer.

It should be also emphasized that there are several limitations of our study and constraints in interpreting results concerning the metallothionein 2A activity and MT2A SNPs in tumorigenesis and neoplastic progression. While the −5 (rs28366003) SNP in the *MT2A* gene could be considered as a potential biomarker for the etiopathology and a higher susceptibility of sinonasal inverted papilloma, as well as an indicator of the dynamics of tumor growth, discrepancies shown in the discussion exist for other neoplastic lesions due to variation of tumor origins, proliferation, and histological differentiation status, which cause differences in their biology. It should be also stressed that the difficulties in obtaining human autopsy materials and using cell culture or laboratory animal material, as well as the diversity of tissues used (fresh tumor samples, cell lines, or blood), may affect the final results of research. Moreover, the relatively small size and heterogeneity of groups studied, the small proportion of homozygote atypical, and variety of analyzed populations can influence the resulting odds ratio estimates and may cause difficulties in the interpretation of results.

Our study provides evidence for implicating MT2A polymorphisms in the development of sinonasal inverted papilloma. The findings also illustrate the key role played by the rs28366003 SNP in the *MT2A* gene in inducing an aggressive Schneiderian papilloma phenotype. These observations provide future possible targets for diagnosis and novel therapeutic strategies for inhibition of tumorigenesis, growth, and progression of this locally aggressive process. However, we realize that our findings represent only one voice in an ongoing discussion and further studies of greater dimensions are required to determine the precise role of MT2A in the carcinogenicity and physiopathology of neoplastic diseases.

## Conclusions

In conclusion, despite the limitation discussed, accumulating evidence indicates that the genetic variation of *MT2A* may contribute to the pathogenesis of sinonasal Schneiderian inverted papilloma in a Polish population. The identification of these tumor-related −5 A/G (rs28366003) *MT2A* gene polymorphisms could also be concerned with the more aggressive local behavior of inverted papilloma cases and used as a potential prognostic biomarker
